# The feasibility of a two-day intensive forest therapy incorporating mindfulness practices for natural disaster-affected individuals: a brief report

**DOI:** 10.3389/fpsyg.2025.1603924

**Published:** 2025-06-20

**Authors:** Yongjun Lee, Yeji Yang, Ji-Eun Pyo, Beom Lee, ChangHyou Kim, Jeong-Ho Choi, Kee-Hong Choi

**Affiliations:** ^1^School of Psychology, Korea University, Seoul, Republic of Korea; ^2^Mind Health KU, Korea University, Seoul, Republic of Korea; ^3^Korea Forest Welfare Institute, Forest Welfare R&D Cneter, Daejeon, Republic of Korea

**Keywords:** forest therapy, natural disaster, psychological distress, mindfulness, stress response, anxiety symptoms, depression symptoms, vitality

## Abstract

This study investigated the feasibility and psychological benefits of a two-day intensive forest therapy program incorporating mindfulness practices for individuals affected by natural disasters. The sample comprised 110 participants, including those who had either experienced wildfires or floods or served as first responders. The results revealed significant reductions in depression, anxiety, and stress pre- and post-intervention, alongside improvements in vitality and mindfulness. Furthermore, enhanced mindfulness significantly moderated reductions in anxiety, stress, and depression. These findings indicate that intensive forest therapy is a viable and promising intervention for improving the mental health of disaster-affected populations.

## 1 Introduction

Climate change has intensified extreme weather events, such as heatwaves, floods, and wildfires, resulting in physical and economic damage and heightened psychological distress (IPCC, 2023). Individuals exposed to such disasters face a higher risk of depression, anxiety, and suicide (Burrows et al., 2024), with posttraumatic stress disorder (PTSD) being particularly prevalent among survivors and first responders (Beaglehole et al., 2018; Herzog et al., 2022). While some stress responses are immediate and transient (Norris et al., 2002), several individuals experience prolonged distress due to insufficient psychosocial support and recovery resources (Guilaran et al., 2018; Rouhanizadeh et al., 2020; Newnham et al., 2022).

Nature-based therapy (NBT), also known as nature-assisted therapy, natural therapy, or green care, encompasses interventions designed to promote physical and mental well-being through engagement with natural environments. Numerous studies have reported the mental health benefits of nature-based therapy, including reductions in stress level (Yao et al., 2021; Johansson et al., 2022), depression (Hyvönen et al., 2023), anxiety (Vitagliano et al., 2023), and improvements in mindfulness (Schutte and Malouff, 2018). Among nature-based therapy, forest therapy, also known as forest bathing or *shinrin-yoku*, has emerged as a promising intervention for alleviating psychological distress (Tsunetsugu et al., 2010; Hartwell et al., 2023). Given that more than half of South Korea’s terrain is mountainous, forest therapy has been widely recognized as a community-based mental health intervention, prompting extensive research on its implementation. Studies have demonstrated its efficacy in reducing depression, anxiety, and stress across diverse populations, including young (Bettmann et al., 2017), middle-aged (Li et al., 2016), and older adults (Lee and Son, 2018) as well as individuals with depressive disorders (Yeon et al., 2023), postmenopausal women (Yu et al., 2016), and those with chronic widespread pain (Han et al., 2016). In particular, forest-therapy has shown notable effects in improving mental health across various stressor-related disorders and symptoms, demonstrating reductions in both physiological and psychological stress levels, as well as benefits in emotional and mood outcomes (Zhang et al., 2023).

Despite its potential, limited research has examined the feasibility of forest therapy for disaster-affected populations. Only a few small-scale studies have investigated its impact on specific occupational groups such as firefighters (Poulsen et al., 2016; Park et al., 2019). To date, there is a lack of studies addressing forest therapy interventions in communities affected by natural disasters, a group known to be susceptible to high level of stress, depression and anxiety (Keya et al., 2023). Additionally, although forest therapy and mindfulness based interventions are distinct concepts, many forest therapy programs incorporate mindfulness elements as a core component (Choi et al., 2024), due to their potential cumulative or synergistic effects when combined (Kaplan, 2001; Schutte and Malouff, 2018). Thus, we explicitly incorporated mindful practices, such as mindful tasting, breath awareness and relaxation techniques, and examined its role in psychological distress reduction.

This study explored the feasibility of a two-day forest therapy program including mindfulness-based practices for individuals affected by natural disasters in reducing depression, anxiety, and in enhancing vitality and mindfulness. Furthermore, we examined mindfulness as a key process variable, specifically its moderating effect.

## 2 Method

### 2.1 Participants

Participants were recruited via advertisements in collaboration with local fire stations, community health centers, and volunteer service centers. Eligibility criteria included individuals who had either experienced wildfire or flood-related damage or served as first responders exhibiting at least mild symptoms of depression or anxiety, defined as a score of 8 or higher on the MHS:D and/or 10 or higher on the MHS:A, respectively. Exclusion criteria comprised physical disabilities that could impede participation in forest therapy activities, such as requiring a wheelchair, significant cognitive impairment or neurological disorders that could hinder questionnaire completion, and severe mental health conditions such as psychosis or suicidal ideation requiring immediate pharmacological intervention or hospitalization. Of the 132 individuals deemed eligible, 22 did not complete the post-assessment, resulting in a final sample of 110 participants for analysis.

### 2.2 Measures

#### 2.2.1 Depressive symptoms

The Mental Health Screening tool for depressive disorders (MHS:D) (Park et al., 2022) is a self-report measure designed to identify individuals in the early stages of Major Depressive Disorder (MDD) with relatively high accuracy in primary care settings. It comprises 12 items rated on a 5-point Likert scale (0 = Not at all, 4 = Extremely), assessing diagnostic symptoms for MDD in the DSM-5, such as depressed mood, diminished interest and pleasure, and psychomotor agitation. A domestic validation study demonstrated high internal consistency (Cronbach’s *α* = 0.940), and in the present study, it was 0.931.

#### 2.2.2 Anxiety symptoms

The Mental Health Screening tool for Generalized Anxiety Disorders (MHS:A) (Kim et al., 2021) is a self-report measure developed to detect individuals in the early stages of Generalized Anxiety Disorder (GAD) with relatively high accuracy in primary care settings. Similarly, it consists of 12 items rated on a 5-point Likert scale (0 = Not at all, 4 = Always), assessing diagnostic symptoms for GAD in the DSM-5, such as excessive anxiety, uncontrollable worry, and restlessness. A domestic validation study reported high internal consistency (Cronbach’s *α* = 0.940) and 0.957 in the present study.

#### 2.2.3 Vitality

The Core Life Activities Scale (CORE) is a self-report measure assessing an individual’s engagement in daily activities (Cho et al., 2024). This scale measures five key life activities: sleeping, eating, physical activity, intimate relationships, and learning new things. It includes five items rated on a 5-point Likert scale (1 = Never, 5 = Always), with higher scores indicating greater engagement in daily life activities. A domestic validation study confirmed the scale’s reliability and validity, with an internal consistency (Cronbach’s *α*) of 0.793.

#### 2.2.4 Mindfulness

The short form of the Five Facet Mindfulness Questionnaire (FFMQ-SF) (Cheong et al., 2017) a condensed and validated version of the original FFMQ (Baer et al., 2004). It comprises five subscales, including acting with awareness, non-judging of experience, observing, non-reactivity, and describing, with three items per subscale totaling 15 items. The original validation study reported an internal consistency of 0.76, whereas the internal consistency in the present study was 0.821.

#### 2.2.5 Stress response

The short form of the Stress Response Scale is a validated adaptation of the original 39-item scale (Koh et al., 2000), later revised by Choi et al. (2006). It consists of three subfactors: somatization, anger, and depression, totaling 22 items rated on a 5-point Likert scale (0 = not at all, 4 = extremely). The original validation study reported a significant internal consistency (Cronbach’s *α* = 0.97) and 0.971 in the present study.

#### 2.2.6 Intervention

The forest therapy programs were developed under the consultation of a multidisciplinary team of experts, including clinical psychologists, certified forest therapy instructors, and disaster relief specialists. The programs were delivered by nationally certified forest therapy instructors. Although they were affiliated with the research team, they were not involved in data analysis and manuscript writing in order to minimize potential bias. In addition, they received sufficient training after the program was developed and participated in weekly meetings to promote consistency in program delivery.

The programs were designed as a two-day, one-night experience. On the first day, participants arrived at the forest welfare facility in the morning and engaged in two forest therapy activities in the afternoon (e.g., mindful tasting, forest mindfulness, or physical activities in a forest setting). On the following morning, they participated in one additional forest therapy activity, completing a total of three distinct forest therapy experiences.

The programs followed a three-phase structure designed to primarily alleviate psychological distress in individuals affected by disasters. Research indicates that disaster survivors experience sensory abnormalities, including heightened arousal and reactivity (Aoki and Nozawa, 2024). In response, the initial *engagement* phase incorporated mindful tasting, where the participants engaged in food preparation, tasting, and savoring to enhance multi-sensory awareness and regulate these sensory symptoms. In the intermediate *rest* phase, forest mindfulness was introduced, including breath awareness and relaxation techniques, to mitigate trauma-related alterations in arousal and reactivity (Eriksen and Ditrich, 2015). The final *integration* phase involved group physical activities in a forest setting aimed at fostering connections between the participants and nature (Newland and Legg, 2024). All research procedures were approved by the local institutional review board.

### 2.3 Statistical analysis

All individual data were analyzed using assigned identification codes to prevent the disclosure of personal information and files containing participants’ names were immediately destroyed upon completion of the study period. Baseline demographic characteristics are presented in Supplementary Table 1. Multilevel analysis was conducted to evaluate whether changes in psychological distress and well-being scores from pre- to post-intervention were statistically significant. This analysis also assessed whether changes in mindfulness moderated pre- to post-scores for anxiety, stress, and depression. In this study, measurement occasion (pre- and post-intervention) was designated as the level-1 parameter, individuals as the level-2 parameter, and program as the level-3 parameter. Additionally, effect sizes were calculated based on the mean differences between the pre- and post-intervention scores.

## 3 Results

Supplementary Table 1 presents the baseline characteristics of the participants, including gender, age, employment status, marital status, education level, and household size. The study included 110 participants, with a mean age of 47.75 years (SD = 11.35). The sample comprised 55.5% females and 41.8% males. In terms of employment, 63.6% were engaged in full-time work. Most participants (71.8%) were married, and over half (67.3%) held a bachelor’s degree. Regarding household composition, four-person households were the most prevalent (34.5%), followed by those with three, two, or five members.

Table 1 presents the effect sizes (Cohen’s *d*), and the results of the multilevel analysis comparing pre- and post-intervention measurements. Statistically significant reductions were observed in depression (B = −6.249, *p* < 0.001) with a medium effect (*d* = 0.68), anxiety (B = −6.457, *p* < 0.001) with a medium effect (*d* = 0.69), and stress (B = −13.952, *p* < 0.001) with a large effect (*d* = 0.87), while significant improvements were found in vitality (B = 3.483, *p* < 0.010) with a medium effect (*d* = 0.77) and mindfulness (B = 4.907, *p* < 0.010) with a small effect (*d* = 0.47).

**Table 1:** Effects sizes and the results of multilevel analysis on five measures.

		Multilevel analysis							Mean scores	
	Cohen’s *d*	Effects estimate	95% CI		*SE*	*df*	*t*	*p*-value	Pre (*N* = 110)	Post (*N* = 110)
			Lower	Upper						
MHS:D	0.68								12.98 (8.21)	6.76 (7.27)
Intercept		19.261	16.280	22.243	1.504	109	12.804	<0.001***		
Time		−6.249	−7.982	−4.515	0.875	108	−7.143	<0.001***		
MHS:A	0.69								15.62 (7.00)	8.94 (8.61)
Intercept		21.871	18.525	25.216	1.688	108	12.958	<0.001***		
Time		−6.457	−8.508	−4.407	1.034	108	−6.243	<0.001***		
CORE	0.77								14.26 (2.83)	18.04 (4.27)
Intercept		10.806	8.867	12.746	0.978	108	11.045	<0.001***		
Time		3.483	2.041	4.924	0.727	108	4.790	0.004**		
FFMQ	0.47								58.94 (9.40)	64.21 (10.81)
Intercept		54.087	49.196	58.978	2.467	108	21.922	<0.001***		
Time		4.907	1.884	7.931	1.525	108	3.217	0.002**		
SRI	0.87								32.12 (17.81)	17.50 (16.65)
Intercept		45.884	38.174	53.595	3.889	106	11.798	<0.001***		
Time		−13.952	−17.873	−10.030	1.978	106	−7.054	<0.001***		

SE, standard error; *df*, degree of freedom; *t*, *t*-value; *p-value < 0.05; **p-value < 0.01; ***p-value < 0.001; MHS:D, Mental Health Screening Tool for Depressive disorders; MHS:A, Mental Health Screening Tool for Anxiety disorders; CORE, Core Life Activities Scale; FFMQ, Five Facet Mindfulness Questionnaire Short Form; SRI, Stress Response Inventory.

Supplementary Table 2 reports the moderating effects of mindfulness on anxiety, stress, and depression. The regression coefficients for the interaction terms (Time × mindfulness) were statistically significant for anxiety (B = −0.446, *p* < 0.001), stress (B = −0.687, *p* = 0.007), and depression (B = −0.377, *p* < 0.001), indicating that mindfulness significantly moderates changes in these variables from pre- to post-intervention.

**Figure 1:**
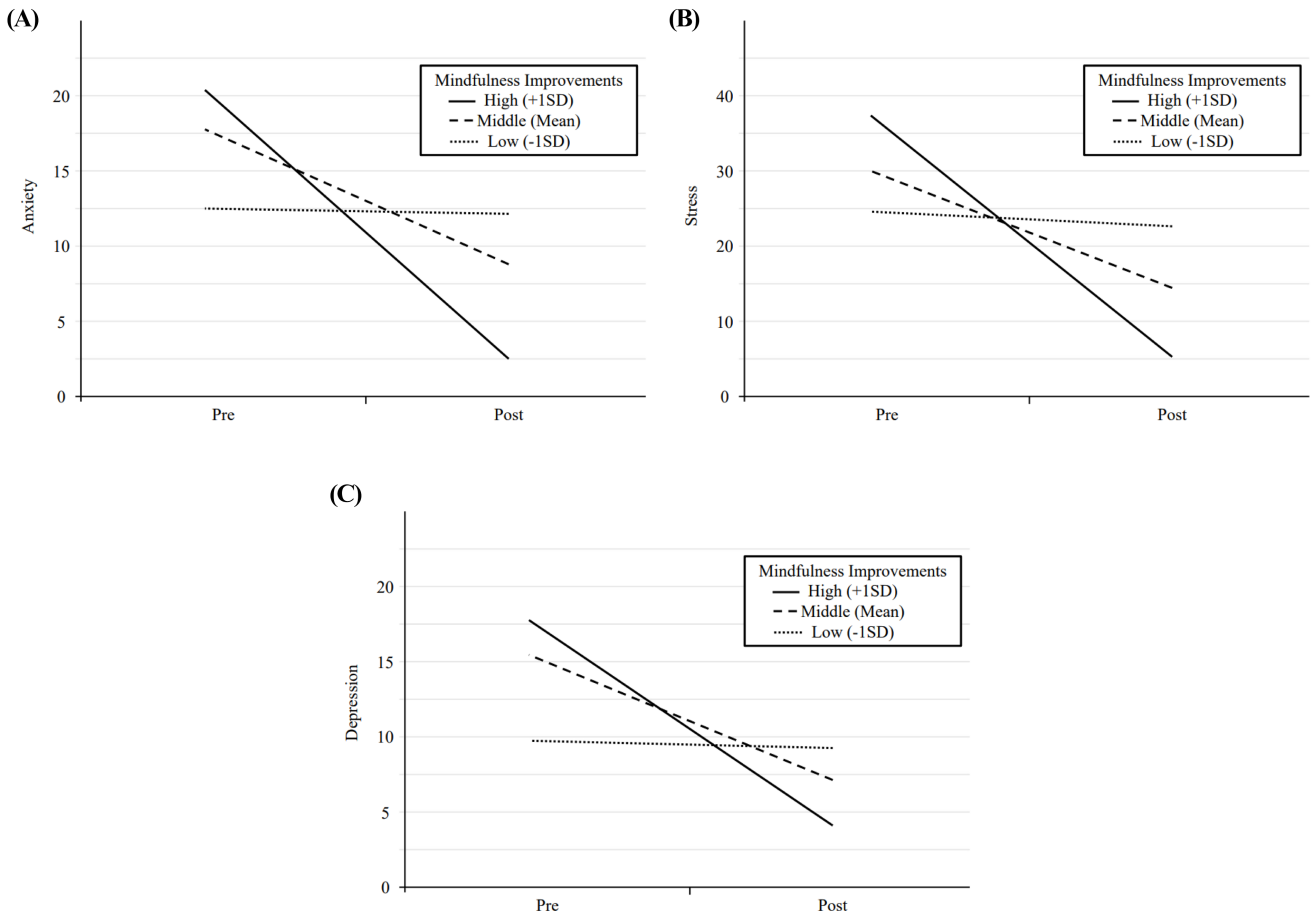
Time × mindfulness across outcome variables. **(A)** Anxiety vs. Time. **(B)** Stress vs. Time. **(C)** Depression vs. Time. Mindfulness improvement levels are represented as follows: solid line for high improvement, dashed line for middle improvement, and dotted line for low improvement.

Figure 1 illustrates the moderating effect of mindfulness on changes in psychological distress from pre- to post-intervention. The solid line represents individuals with high mindfulness (+1 SD), the dashed line represents the mean mindfulness level, and the dotted line represents individuals with low mindfulness (−1 SD). The figure demonstrates that participants with greater improvements in mindfulness exhibited significant reductions in anxiety, stress, and depression over time.

## 4 Discussion

This study examined the feasibility and effectiveness of intensive forest therapy in reducing psychological distress and enhancing well-being among individuals affected by natural disasters. Additionally, it incorporated mindfulness practices into the forest therapy programs and investigated the role of mindfulness in psychological distress reduction. The findings revealed that participation in a two-day intensive forest therapy program is assumed to reduce in depression, anxiety, and stress and promote improvements in vitality and mindfulness. Notably, participants who exhibited greater improvements in mindfulness revealed greater reductions in anxiety, stress, and depression. These results align with previous studies, suggesting that forest therapy could be a feasible and promising intervention for improving the mental health of disaster-affected populations (Yeon et al., 2021).

Notably, this intervention seems to hold particular significance for individuals with low socioeconomic status, who are disproportionately affected by natural disasters (Mao and Agyapong, 2021) and often face barriers to accessing effective treatment due to time and financial constraints (Niemeyer and Knaevelsrud, 2023). Forest therapy may serve as a readily accessible option, particularly in South Korea, where forests are widely and easily accessible. Preliminary findings within just 2 days suggests its potential as a feasible intervention for underserved populations with limited access to conventional mental health care.

Further analysis revealed that improvements in mindfulness significantly moderated reductions in anxiety, stress, and depression. These results likely stem from the integration of multi-sensory awareness and relaxation techniques, highlighting the potential of mindfulness as a core component in nature-based interventions (Schutte and Malouff, 2018).

Even though forest therapy is a nature-based intervention rather than an intensive psychological treatment like other evidence-based approaches (e.g., trauma-focused CBT), the study’s dropout rate of 16% was comparable to or lower than the rates reported in previous studies, including 21% in PTSD treatment (Varker et al., 2021), 22% in a brief intervention for disaster-exposed service providers (Powell et al., 2022), and 13% in EMDR for earthquake survivors (Saltini et al., 2018). Thus, it may be feasible to provide nature-based treatments as adjuncts to evidence-based psychological interventions.

In summary, a two-day intensive forest therapy program appears to be effective in reducing depression, anxiety, and stress levels while enhancing vitality and mindfulness among natural disaster-affected populations. Furthermore, mindfulness emerged as a key process variable, significantly moderating reductions in anxiety, stress, and depression. These findings provide preliminary empirical support for the applicability of forest therapy in disaster contexts and lay the basis for future controlled trials to evaluate its efficacy and underlying mechanisms.

This study has several limitations. First, the lack of a control group and follow-up assessments makes it difficult to draw definite conclusions about the efficacy and durability of the forest therapy, underscoring the need for replication in future randomized controlled studies with larger samples and follow-up assessments. Second, as the present study relied on self-report measures to examine intervention effects, incorporating physiological indicators such as cortisol levels in future research may strengthen the validity of the findings. Lastly, since the study was conducted only with Korean samples affected by natural disasters, further research is needed to determine whether the results are generalizable to clinically diagnosed PTSD patients or other populations. Despite these limitations, this study contributes to the growing body of literature on nature-based interventions, offering promising evidence of their applicability in disaster contexts.

## Data Availability

The raw data supporting the conclusions of this article will be made available by the authors, without undue reservation.
