# Supplementary material for: The feasibility of a two-day intensive forest therapy incorporating mindfulness practices for natural disaster-affected individuals: a brief report
Source: Front Psychol. 2025 Jun 20;16:1603924. doi: 10.3389/fpsyg.2025.1603924 (PMC12226552; doi:10.3389/fpsyg.2025.1603924)
Supplement: Supplementary file 1 [file Supplementary_file_1.docx]

Supplementary Material

# Supplementary Data

# Supplementary Figures and Tables

## Supplementary Figures

## Supplementary Tables

**Supplementary Table 1.** Baseline demographics of participants.

| **Baseline Demographics** | **N (%)** |
| --- | --- |
| **Gender,** n (%) |  |
| Male | 46 (41.8) |
| Female | 61 (55.5) |
| Unknown | 3 (2.7) |
| **Age,** M (SD) | 47.75 (11.35) |
| **Employment,** n (%) |  |
| Unemployed | 3 (2.7) |
| Retired | 7 (6.4) |
| Student | 1 (0.9) |
| Homemaker | 17 (15.5) |
| Part-time Employed | 8 (7.3) |
| Full-time Employed | 70 (63.6) |
| Unknown | 4 (3.6) |
| **Marital Status,** n (%) |  |
| Never Married | 21 (19.1) |
| Married | 79 (71.8) |
| Divorced | 1 (0.9) |
| Widowed | 5 (4.5) |
| Unknown | 4 (3.6) |
| **Education,** n (%) |  |
| High School (≤ 12 years) | 28 (25.5) |
| College Bachelor’s degree (≤ 14 years) | 24 (21.8) |
| University Bachelor’s degree (≤ 16 years) | 50 (45.5) |
| Higher Education (> 16 years) | 5 (4.5) |
| Unknown | 3 (2.7) |
| **Household size,** n (%) |  |
| Single-person Household | 11 (10.0) |
| Two-person Households | 19 (17.3) |
| Three-person Households | 25 (22.7) |
| Four-person Households | 38 (34.5) |
| Five-person Households | 13 (11.8) |
| Unknown | 4 (3.6) |

**Supplementary Table 2.** The results of the moderation analysis of mindfulness on anxiety, stress, and depression

|  | **Effects Estimate** | **95% CI** | | ***SE*** | ***df*** | ***t*** | ***p*-value** |
| --- | --- | --- | --- | --- | --- | --- | --- |
|  |  | **Lower** | **Upper** |  |  |  |  |
| **MHS:A** |  |  |  |  |  |  |  |
| Intercept | 22.032 | 19.630 | 24.434 | 1.212 | 107 | 18.183 | $<$.001*** |
| Time | $-$6.462 | $-$8.044 | $-$4.880 | 0.798 | 107 | $-$8.097 | $<$.001*** |
| Mindfulness | 0.694 | 0.497 | 0.891 | 0.099 | 93 | 7.010 | $<$.001*** |
| Time$\times$Mindfulness | $-$0.446 | $-$0.586 | $-$0.305 | 0.071 | 107 | $-$6.289 | $<$.001*** |
| **SRI** |  |  |  |  |  |  |  |
| Intercept | 46.453 | 39.986 | 52.920 | 3.261 | 105 | 14.243 | $<$.001*** |
| Time | $-$14.204 | $-$17.205 | $-$11.203 | 1.514 | 105 | $-$9.385 | $<$.001*** |
| Mindfulness | 1.014 | 0.569 | 1.459 | 0.224 | 93 | 4.528 | $.$005** |
| Time$\times$Mindfulness | $-$0.687 | $-$0.936 | $-$0.438 | 0.126 | 105 | $-$5.465 | .007** |
| **MHS:D** |  |  |  |  |  |  |  |
| Intercept | 19.138 | 16.544 | 21.733 | 1.309 | 107 | 14.623 | $<$.001*** |
| Time | $-$6.123 | $-$7.634 | $-$4.613 | 0.762 | 107 | $-$8.037 | $<$.001*** |
| Mindfulness | 0.639 | 0.423 | 0.854 | 0.108 | 93 | 5.891 | $<$.001*** |
| Time$\times$Mindfulness | $-$0.377 | $-$0.509 | $-$0.245 | 0.066 | 107 | $-$5.666 | $<$.001*** |

Note: *SE*, standard error; *df*, degree of freedom; *t*, t-value; * *p*-value < 0.05; ***p*-value < 0.01; ****p*-value < 0.001; MHS:A, Mental Health Screening Tool for Anxiety disorders; SRI, Stress Response Inventory; MHS:D, Mental Health Screening Tool for Depressive disorders.
